# Role of Avacopan on Antineutrophil Cytoplasmic Antibody (ANCA)-Associated Vasculitis

**DOI:** 10.3390/jcm13226676

**Published:** 2024-11-07

**Authors:** Justo Sandino, Enrique Morales

**Affiliations:** 1 Department of Nephrology, Hospital 12 de Octubre, 28041 Madrid, Spain; justosandino14@gmail.com; 2 Research Institute of University Hospital 12 de Octubre (imas12), Department of Medicine, Complutense University of Madrid, 28041 Madrid, Spain

**Keywords:** vasculitis, glucocorticoids, avacopan

## Abstract

Antineutrophil cytoplasmic antibody-associated (ANCA) vasculitis are a group of autoimmune diseases characterized by inflammation of the microvasculature, leading to life-threatening complications, including kidney disease. These diseases are associated with a high morbidity and mortality rate. Conventional treatment modalities have evolved towards personalized therapies intending to mitigate inflammation and minimize the adverse effects of traditional immunosuppressive agents. Avacopan, a novel C5a receptor inhibitor, represents a promising therapeutic option for vasculitis with renal involvement. This article provides a comprehensive review of the role of complement in the pathogenesis of vasculitis with renal involvement and the role of avacopan for its treatment, taking into account recent updates to both the EULAR and KDIGO guidelines and published experience of avacopan use in real clinical settings.

## 1. Introduction

Antineutrophil cytoplasmic antibody-associated (ANCA)-associated vasculitis (AAV) encompasses a spectrum of diseases characterized by small vessel inflammation. These include granulomatosis with polyangiitis (GPA), microscopic polyangiitis (MPA), and eosinophilic granulomatosis with polyangiitis (EGPA). The incidence of AAV has increased over the past decades, potentially due to increased awareness among clinicians and the wider availability of ANCA testing. However, the majority of data pertaining to incidence and prevalence originates from registries in Europe, Australia, Japan, and the USA, with a limited representation from Latin America, Africa, and the remainder of Asia. Incidence varies between 24.7 per million in Norway and 33.0 per million in the USA, with MPA being more prevalent than GPA in China and Japan, whereas EGPA was identified as the rarest form of AAV across all countries. A global study reported that MPO-ANCA was significantly more prevalent among individuals of Japanese, Chinese, and Southern European descent than among those of Northern European descent [1].

In studies examining data from the late twentieth century, the age-specific incidence was highest among individuals aged 65–74 years in the UK and Finland, however, data from early twenty-first century in the UK, show that the age of the peak incidence of AAV has increased to over 80 years of age. At the opposite end of the age spectrum, AAV is exceedingly rare. AAV is generally slightly more prevalent in males than in females, with a male-to-female ratio of between 1.07:1 and 1.48:1. To date, no definitive infectious trigger has been identified for AAV. Furthermore, the potential role of seasonality as a contributing factor to infection has been explored; however, this has not revealed any discernible pattern. European studies in the 1990s indicated a potential increase in GPA during the winter season. However, more recent studies have even proposed a correlation with summertime, or have identified no association between GPA and seasonality. Other risk factors that have been reported include rural living and farming, with no definitive correlation with socio-economic status confirmed thus far [2].

The treatment of vasculitis with renal involvement entails the administration of high-dose glucocorticoids alongside other immunosuppressive agents, including cyclophosphamide, azathioprine, and monoclonal antibodies such as rituximab. This approach has been employed to achieve disease remission and maintain said remission through the prevention of relapse [3]. Despite their proven efficacy, these drugs are associated with a wide range of adverse effects, including infections, neoplasms, and cardiovascular events, collectively known as cumulative organ damage [4]. Furthermore, a considerable number of patients experience disease relapse or are unresponsive to conventional treatment [5]. This has prompted the development of alternative therapeutic strategies.

In this context, avacopan, an oral molecule able to inhibit the C5a receptor of the alternative complement pathway, has emerged as a promising therapeutic option for treating vasculitis with renal involvement. By acting on the terminal part of the complement cascade, avacopan results in potent anti-inflammatory effects while avoiding a significant cumulative immunosuppression burden [6,7]. This paper provides a well-organized explanation of the role of avacopan in the treatment of AAV with renal involvement, focusing on the mechanism of action of avacopan, the role of the complement cascade, and comparing its clinical efficacy to that of conventional treatments, with particular interest in published experience with avacopan in real clinical settings.

## 2. The Role of Complement on AAV

**Figure 1:**
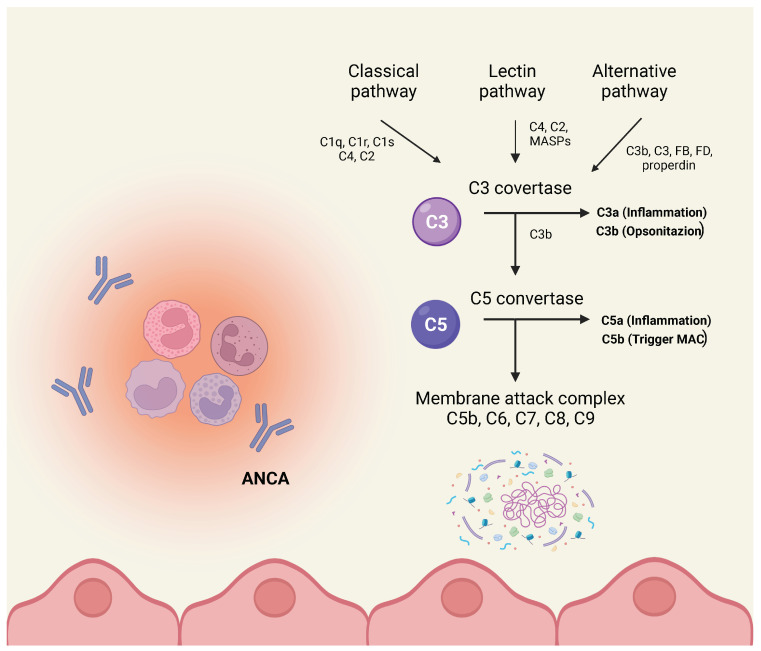
Different pathways of complement activation.

Complement pathways and immune complex formation are central to the pathogenesis of AAV (Figure 1), although the exact mechanism and site of activation still need to be better understood [5]. To address this matter, several studies were conducted in complement-depleted murine models of AAV by administration of cobra venom factor (a potent complement-depleting agent), showing that mice pre-injected with complement factor did not develop glomerular injury following AAV generation, whereas mice that did not receive the venom inevitably developed the expected renal injury [8].

To further characterize the pathway and site of complement activation involved in this process, renal vasculitis was then generated using the same passive immunization procedure in C4-depleted knock-out mice (C4 -/-), C5-depleted knock-out mice (C5 -/-), and a factor B-deficient knock-out model (FB -/-). It was observed that none of the C5-deficient and factor B-deficient mice developed the disease, whereas all the C4-deficient knock-out mice developed glomerulonephritis. Both the classic and lectin pathways require activation of C4 to produce C3 and C5 convertase. C4 -/- mice are unable to generate these convertases and thus cannot activate C3 or C5, suggesting that classical and lectin pathways are not relevant for the development of the glomerular lesions characteristic of AAV. In contrast, both C5 (a common step for the classical, alternative, and lectin pathways) and factor B depletion (necessary for the activation of the alternative pathway [AP]) are required for the development of renal lesions. This observation proves that ANCA disease does not involve activation of complement via the classic or alternative pathways [8].

Local complement activation in the kidney can be generated at different levels. For example, it is known that the glomerular basement membrane does not express complement regulatory proteins and is dependent on circulating factor H (FH) to control the AP [9]; thus, genetic defects, antibodies, or other proteins may interfere with FH function in this context [10,11].

It has been postulated that podocytes have evolved protective mechanisms to escape complement attack, such as autophagy, endocytosis, and expression of complement regulatory factors. In turn, podocytes express CR1, a receptor that can regulate both the classical and AP. The loss of CR1 may allow complement activation at the podocyte level or may be a consequence of the formation of the membrane attack complex (C5b-9). These findings underscore the potential for targeted interventions that enhance podocyte protective mechanisms or restore CR1 function [12].

There is minimal expression of complement regulatory proteins on the surface of tubular epithelial cells [13]. This compartment is not generally in contact with complement proteins, but AP activation may cause tubulointerstitial injury in proteinuric nephropathies due to the passage of complement proteins into the tubules [14]. Tubular epithelial cells also synthesize C3, and this local production may be responsible for acute or chronic tubulointerstitial injury [15,16].

It has been proposed that baseline serum C3 levels have independent prognostic value in predicting long-term renal and overall survival in patients with renal vasculitis, with a worse prognosis in those patients with decreased C3 levels at diagnosis [17].

In patients with active AAV, urine and plasma levels of C3a, Bb, C5a, and sC5b-9 are significantly higher than those in remission [18]. Several studies have shown that deposition of certain complement elements (C3, C4, C1q, factor B, properdin, and CAM) in renal biopsies from patients with vasculitis is associated with increased proteinuria and worse renal function [19,20]. In this regard, low levels of C3 are associated with a more aggressive histological presentation of vasculitis and a worse renal prognosis in several models. On the other hand, C3d and properdin deposits in renal biopsy, suggesting activation of complement AP, have been correlated with a higher percentage of crescents and proteinuria [21]. Thrombotic microangiopathy in renal biopsy associated with vasculitis is not uncommon (between 13 and 15%), especially in severe cases with a worse course [22].

Protective genetic variants in components of the AP against the development of AAV (CFB32Q/W) have been described, as well as other variants that are associated with severe forms of the disease (CFH-H1, CFH-H2, and ΔCFHR3/1) [23].

## 3. Avacopan for Treating AAV: The ADVOCATE Trial as Starting Point

Avacopan is a molecule available in an oral formulation capable of inhibiting the activation of complement AP through selective binding to the C5a receptor (C5aR). The C5a fraction is a potent anaphylotoxin generated upon activation of the complement cascade and exerts its effects upon interaction with its specific receptor, which is expressed in numerous cells, including neutrophils, monocytes, and macrophages. The binding of C5a to its receptor triggers intracellular signaling cascades, activating inflammatory mediators, cytokine release, and subsequent amplifying of the immune response. Avacopan can selectively bind to C5aR with very high affinity, selectively blocking the inflammatory effects mediated by this pathway without interfering with the formation of the membrane attack complex [24].

Several preclinical studies have demonstrated the drug’s efficacy in models of patients with ANCA-associated vasculitis [6,7], leading to the development of ADVOCATE (A Study to Compare the Safety and Efficacy of Avacopan to Standard Prednisone Treatment in Subjects with ANCA-Associated Vasculitis), a multinational, randomized, double-masked, controlled trial evaluating the efficacy and safety of avacopan in combination with rituximab or cyclophosphamide as induction therapy in patients with AAV. The study’s primary objective was to assess the proportion of patients who achieved remission, defined as a Birmingham Vasculitis Activity Score (BVAS) of 0 or complete withdrawal of glucocorticoids, after 26 weeks of treatment.

The results of the ADVOCATE study show that avacopan is not inferior to standard glucocorticoid therapy in achieving remission in patients with AAV. The avacopan group received significantly lower doses of glucocorticoids and also experienced fewer glucocorticoid-related adverse events. Importantly, the safety profile of avacopan was favorable, with a lower incidence of serious adverse events, infections and cardiovascular events observed in the avacopan group compared to the glucocorticoid group, providing reassurance about its safety profile [25].

For all these reasons, the ADVOCATE trial is a pivotal study in treating vasculitis with renal involvement, providing robust evidence of the efficacy and safety of avacopan in achieving remission in patients with ANCA-associated vasculitis. However, its potential role in disease relapse patients, subjects with low estimate glomerular filtration rates (eGFR), and other sceneries like diffuse alveolar hemorrhage (DAH) remained unexplored in this landmark study.

## 4. What Is There Beyond ADVOCATE?

Data from the ADVOCATE trial have led to modifications in the most recent guidelines for the management of AAV. These guidelines incorporate the use of avacopan as a remission-inducing agent, especially in frail and elderly patients, to avoid the adverse effects of prolonged glucocorticoid use [26].

Following the incorporation of avacopan into current treatment guidelines, several publications describe different experiences with the drug in real clinical settings. A multicenter retrospective study in the US evaluating 92 patients receiving avacopan for the treatment of new-onset or relapsed AAV corroborated the results obtained in the ADVOCATE trial: clinical remission was achieved at week 26 and week 52 in 61 out of 68 patients, (90%) and 32 out of 38 patients (84%), respectively. In patients with renal involvement and follow-up at week 26 or 52, the mean change in estimated glomerular filtration rate (eGFR) from baseline to week 26 and week 52 was +12.2 and +19.8 mL/min per 1.73 m^2^, respectively.

Within the methodological design of the study, patients were stratified according to the time of avacopan initiation (within 30 days or after 30 days of induction therapy initiation). Patients who received avacopan within 30 days of induction therapy were more likely to have a higher BVAS at baseline (8.3 vs. 4.4) (*p* < 0.01) and less likely to require plasmapheresis at baseline (7% vs. 24%) (*p* = 0.05). There was also a trend towards more rituximab use (56% vs. 35%) and fewer glucocorticoid pulse doses (56% vs. 76%) in those who received avacopan within the first 30 days compared to those who received it late, respectively.

Patients who started avacopan ≥ 30 days from induction treatment, compared with <30 days, had a higher mean cumulative prednisone dose at both week 12 [2158 (996) mg vs. 1501 (1141) mg (*p* = 0.03), respectively] and week 52 (2723 (1398) mg vs. 1787 (1610) mg [*p* = 0.03], respectively). Similarly, patients who received avacopan before day 30 were more likely to discontinue steroids (compared to day 30 or later), with a probability of stopping steroids at week 26 of 52% (vs. 33%) and week 52 of 20% (vs. 19%) in both groups, respectively. In patients with renal involvement (*n* = 71), those who started avacopan ≥ 30 days after initiation of induction therapy took longer to reach nadir proteinuria: 173 (100–260) days versus 84 (43–142) days (*p* = 0.01).

Among the strengths of this study are the inclusion of patients with eGFR less than 15 mL/min per 1.73 m^2^, dialysis-dependent patients, and patients receiving plasmapheresis within induction therapy. No adverse effects due to renal toxicity or alterations in plasma concentrations after dialysis sessions were reported (due to the molecular weight of avacopan, high protein affinity, and predominantly hepatic elimination). However, in this study, higher cumulative doses of glucocorticoids are administered compared to the ADVOCATE trial; the longer delay in starting avacopan explains this significant difference, with a median of three weeks passed after the start of induction therapy.

On the other hand, the safety profile of avacopan was the same as that reported in ADVOCATE, with hepatotoxicity being the most common reason for discontinuation [27]. However, some case reports suggest that concomitant use of ursodeoxycholic acid may mitigate avacopan-induced hepatotoxicity [28]. Other less common adverse reactions include hypersensitivity, angioedema, diarrhea, neutropenia, lymphopenia, and thrombopenia within the first week of treatment, with improvement after three weeks of discontinuation [29].

More data on the use of avacopan in patients with eGFR of less than 15 mL/min/1.73 m^2^ show that avacopan is safe and effective in this group of patients, as shown in a small cohort of four cases in which treatment with avacopan was tested, with no serious adverse events observed. Glucocorticoids were rapidly tapered after initiation of avacopan, and the drug was maintained for at least 12 months. All patients experienced substantial recovery of eGFR despite profound renal dysfunction at presentation [30].

Some factors like older age and lower eGFR at diagnosis are associated with a poor prognosis in patients with AAV [31]. On this line, a post hoc study of the ADVOCATE trial in a subgroup of patients with eGFR of less than 20 mL/min/1.73 m^2^ showed that patients treated with avacopan had a significantly higher recovery of eGFR after 52 weeks of treatment compared to the group treated with glucocorticoids alone (16.1 vs. 7.7 mL/min/1.73 m^2^; *p*: 0.003) [32]. Also, a German multicentric retrospective study of 39 patients with AAV reflects their experience with avacopan after a median follow-up time of 41 weeks. Interestingly, higher remission rates were seen at 6 months and sustained remission at 12 months compared with the ADVOCATE trial, including patients with lower eGFR and DAH. Nevertheless, patients who experienced sustained remission were on higher doses of glucocorticoids; this reflects on the higher cumulative dose of prednisolone at week 52 compared with the cumulative dose used on the ADVOCATE trial (3090 mg vs. 2002.9 mg); despite this, no serious adverse effects were reported [33].

Data from an observational study from Spain about the experience with avacopan in 29 patients with AAV (mostly MPO positive) confirm its efficiency as a steroid-sparing agent when compared with a historical cohort that did not receive treatment with avacopan. Subjects treated with avacopan received a significantly lower cumulative prednisone dose at 6 and 12 months (*p*-values of 0.02 and <0.01, respectively) than historical controls. Medium baseline eGFR was 22 mL/min/1.73 m^2^, which reflects that this study included patients with very low eGFR. During follow-up, eGFR increased from a mean of 23.2 ± 11.2 to 38.38 ± 18.55 mL/min after 12 months of diagnosis, with a mean change of 13.69 ± 11.06 mL/min after 12 months of avacopan initiation. Nevertheless, the evolution of eGFR at one year of follow-up and the incidence of relapse were similar in both groups [34] (Table 1).

Analysis of the efficacy and safety of avacopan was performed on patients who received rituximab as induction therapy in the ADVOCATE study. Patients treated with avacopan experienced a significant reduction in disease activity parameters and a reduction in the need for high-dose glucocorticoids. An improvement in quality of life scales was also observed in patients treated with avacopan. Regarding safety, avacopan was well tolerated, and no treatment-related severe adverse events were observed. These results suggest that avacopan may be a promising treatment option for patients with AAV receiving rituximab [37].

Patients with AAV and severe pulmonary involvement in the form of DAH were excluded from the ADVOCATE TRIAL. Nevertheless, avacopan has been successfully used in a series of 15 Mayo Clinic patients with high remission rates after a median of 17 weeks of follow-up [38]. In addition, a small case series of eight patients with AAV and severe pulmonary involvement requiring mechanical ventilation, for whom treatment with avacopan was tested, showed that all patients had a favorable respiratory and renal outcome after drug introduction [39].

The use of avacopan has been tested in other settings than induction therapy for AAV. There is published experience with avacopan in patients who have relapsed after induction therapy with conventional agents, achieving sustained remission after avacopan introduction, regardless of the initial agent used to achieve the first remission [40].

Current and future trends should be towards sparing and even rapid withdrawal of glucocorticoids due to their known adverse effects. In this regard, Patel et al. performed a post hoc analysis of the ADVOCATE trial data. They determined the Glucocorticoid Toxicity Index-Metabolic Domains (GTI-M) from the original GTI, emphasizing metabolic parameters. The usefulness of GTI-M for assessing the metabolic burden associated with glucocorticoid treatment was evaluated. The GTI-M effectively identified adverse metabolic effects of glucocorticoids, including weight gain, dyslipidemia, and glucose intolerance. It also provided clinicians with a concise but comprehensive assessment tool to monitor metabolic toxicity, allowing timely interventions to mitigate risks [41].

Another parameter studied by this same group was the perception of quality of life, finding a significant improvement in quality of life scale parameters (both physical and mental) among patients receiving avacopan compared to those receiving only standard treatment [35].

## 5. Knowledge Gaps

### 5.1. Which Patients May Benefit from Treatment with Avacopan?

The main principles in the management of patients with AAV are based not only on immunosuppression but also on the management of cardiovascular risk factors such as diabetes, dyslipidemia, hypertension, osteoporosis, and other treatment-related complications, always taking into account vulnerable demographic groups such as the elderly, who are more susceptible to higher rates of infection and treatment-related toxicities, and younger patients of reproductive age [42].

Therefore, if we could establish a profile of patients who might benefit from avacopan treatment, we would need to identify those in whom glucocorticoids are contraindicated or who are at high risk of toxicity (frail, elderly, obese, or patients with significant immunosuppressive burden). Tools such as the GTI and GTI-M [41] are available to help stratify the risk of prolonged steroid use in patients with AAV. On the other hand, given the potential benefit demonstrated in various studies [27,30,32,33,34,39] in patients with poor renal function (estimated glomerular filtration rate less than 15 mill/min/1.73 m^2^) who are treated with avacopan, it is thought that this group of patients would be excellent candidates for this treatment. In addition, it has recently been recommended to trial treatment with avacopan in patients who experience relapses or who are refractory to conventional treatment [26,43].

### 5.2. Duration of Treatment and Glucocorticoid Tapering Regime

In AAV treatment, the introduction of avacopan has marked a significant paradigm shift. However, our understanding has crucial gaps, particularly in determining the ideal treatment duration. While the KDIGO guidelines recommend a treatment period of 2–4 years for AAV with conventional therapy [36], there needs to be more evidence on the duration of avacopan treatment. The determination of this ideal duration is of utmost importance, as it directly impacts the prevention of relapse, and the management of risks associated with prolonged immunosuppression and medication-related adverse effects [44].

Regarding the ideal glucocorticoid tapering schedule, the aforementioned study by Zonozi et al. reported a median of 40 days from initiation of avacopan to discontinuation of steroids [27], while the Spanish group reported a median of 56 days to steroid withdrawal on newly diagnosed patients. A French cohort of 31 patients with AAV and contraindication for high dose corticoid therapy who received treatment with avacopan also showed favorable outcomes despite a rapid withdrawal of glucocorticoids in 28 patients, who were steroid-free after two months [45]. The current recommendation for glucocorticoid tapering is to aim for discontinuation four weeks after initiation of avacopan, guiding the glucocorticoid taper regime based on the data derived from the PEXIVAS trial [26,34,46].

### 5.3. Other Considerations

There is no evidence in the renal transplant population with relapsing disease, or about avacopan usefulness in extra-renal predominant manifestations (although there is a case report of granulomatous otitis media in the setting of AAV-MPO that was successfully treated with avacopan without the use of glucocorticoids [47]).

One of the significant knowledge gaps in the treatment of AAV with avacopan is the relationship between the kinetics of ANCA titers and overall patient improvement. Evidence shows that improvement in clinical parameters does not correlate with a decrease in ANCA titer [48]. However, there are no studies with a rigorous methodological design to evaluate this issue.

The heterogeneous nature of AAV and the variability in patient response to avacopan highlight the need for personalized treatment algorithms. Real-world and long-term follow-up studies are essential to assess the efficacy and safety of avacopan beyond controlled clinical settings.

## 6. Conclusions

Treatment modalities for AAV have evolved from non-specific immunosuppression to therapies aimed at reducing inflammation while minimizing treatment-related toxicity. Avacopan, a novel oral small-molecule inhibitor of the complement receptor C5a, has emerged as a promising therapeutic option for treating renal vasculitis. It offers the potential to achieve potent anti-inflammatory effects while preserving host defenses and minimizing systemic immunosuppression.

The ADVOCATE study demonstrated the efficacy and safety of avacopan in inducing remission in patients with ANCA-associated vasculitis. Its safety profile was favorable compared to standard glucocorticoid therapy.

Avacopan may be especially beneficial for patients at high risk of glucocorticoid-related adverse events, patients with a high burden of cardio-metabolic disease, and patients with low eGFR at presentation. Ongoing research is focused on elucidating the long-term safety and efficacy of avacopan in renal clinical settings and exploring its role in maintenance therapy and combination strategies, including its utility outside the setting of AAV.

## Figures and Tables

**Table 1:** Clinical characteristics and outcomes of AAV patients treated with avacopan in the literature.

Author (Ref.)	Population	Time of Avacopan Onset	eGFR at Avacopan Onset	Proteinuria at Avacopan Onset	Renal Outcomes	Follow-Up, weeks	GC Dose at Follow-Up	Adverse Events
Jayne et al. [4]	67 patients with newly diagnosed or relapsing AAV (22 on avacopan vs. 23 controls)	Avacopan initiated at diagnosis or relapse	Baseline eGFR: ~50 mL/min/1.73 m^2^	~0.2–0.3 g/24 h	Improvement in eGFR, reduced proteinuria, better renal survival	12	-	Lower incidence of AE related to GC use on patients treated with avacopan. 15 patients discontinued avacopan due to AE
Merkel et al. [5]	42 patients with newly diagnosed or relapsing AAV	Initiated at diagnosis/relapse	Median eGFR: ~60 mL/min/1.73 m^2^	~0.1 g/24 h	Renal recovery observed, but main focus was on safety	12	10 mg/day by week 11	No differences among treatment groups. Avacopan was discontinued in 4 patients (14%) due to AE
Jayne et al. [23]	331 patients with active AAV, 81% with renal involvement	Avacopan initiated at diagnosis or relapse	Mean baseline eGFR: 44 mL/min/1.73 m^2^	~0.4 g/24 h	Significant eGFR improvement by week 26, sustained through week 52	52	-	Lower incidence of AE related to GC use on patients treated with avacopan. One patient discontinued avacopan due to angioedema, 9 patients suspended avacopan due to abnormal liver function
Leeuwen et al. [35]	8 patients with AAV treated with avacopan	Variable	Not specified	Not specified	eGFR improvement and proteinuria reduction allowing to stop HD	48–96	5 discontinued PDN, 3 on low-dose prednisone	Improvement of steroid-related toxicity was observed. One patient suspended avacopan because of pregnancy wish
Zonozi et al. [25]	92 patients with AAV on avacopan treatment 21 patients with eGFR < 15 mL/min/1.73 m^2^	3.6 (2.1–7.7) weeks after start of induction of remission therapy	43.5 mL/min/1.73 m^2^ (SD 34.2)	1.7 g/24 h (IQR 0.4–2.7)	Clinical remission was achieved in 90% of patients at week 26 and 84% of patients at week 52	52	1.8 mg (3.7) at week 26 0.6 mg (2.5) at week 52	Avacopan was stopped in 30 (33%) patients due to AE: transaminitis, gastrointestinal symptoms, worsening kidney function, and financial cost burden
Gabilan et al. [36]	31 AAV patients, 30 with kidney involvement	20 days (IQR 12; 35)	24 mL/min/1.73 m^2^	1.2 gr/gr	Rapid GC tapering, increase in GFR independently of kidney biopsies findings	48	Rapid GC taper at month 3	2 patients developed acute hepatitis and ARMD, 5 patients developed bacterial infections (avacopan was withdrawn)
Barr et al. [28]	4 patients with AAV and eGFR < 15 mL/min/1.73 m^2^	Pt 1: 19 days Pt 2: 14 days Pt 3: 21 days Pt 4: 28 days	Pt 1: 8 mL/min/1.73 m^2^ Pt 2: 10 mL/min/1.73 m^2^ Pt 3: 7 mL/min/1.73 m^2^ Pt 4: 5 mL/min/1.73 m^2^	Pt 1:128.8 gr/gr Pt 2: 103 gr/gr Pt 3: - Pt 4: 114 gr/gr	All patients experienced substantial eGFR recovery despite profound kidney dysfunction at presentation.	36–52	Pt 1: No GC Pt 2: No GC Pt 3: PDN 5 mg Pt 4: No GC	None reported. Pt 4 suspended avacopan due to no recovery of kidney function
Falde et al. [34]	15 patients with AAV and DAH	18 days (IQR 2–24 days) after starting standard remission induction therapy	Not specified	Not specified	Variable, 2 patients required renal replacement therapy, neither had renal recovery	17 (IQR 6–37)	4 patients remained on GC, the rest were tapered off	Avacopan was discontinued in 6 patients, 2 patients developed serious infections
Zimmermann et al. [31]	39 AAV patients 15 patients (38%) with eGFR < 15 mL/min/1.73 m^2^ 7 patients (18%) requiring dialysis. 7 patients with DAH (18%)	Avacopan initiated at diagnosis or relapse	37 mL/min/1.73 m^2^	1.46 g/g	Remission was achieved in 87.5% at 6 months and sustained remission in 91% (21/23) at 12 months.	41 (12–56)	At month 12, 8 of 23 patients were still on prednisolone (1.25–5 mg)	Avacopan was discontinued due to AEs in 21% of cases, but no serious AEs were reported.
Draibe et al. [32]	29 patients with AAV, 27 with kidney involvement, included patients with eGFR < 15 mL/min/1.73 m^2^	48 days (IQR 19.5–93.5)	23.22 ± 11.26 mL/min/1.73 m^2^	239.54 ± 219.13 g/mol	eGFR increased, with a mean change of 13.69 ± 11.06 mL/min after 12 months of avacopan initiation. Proteinuria decreased from 239.5 ± 219.1 g/mol to 61.4 ± 51 g/mol after 12 months of avacopan treatment.	456.8± 181.7 days	18 patients (60.06%) discontinued GC	13 infections, 2 severe neutropenia (1 death related to neutropenia) At the end of the study 10 patients discontinued avacopan, 7 patients without any particular cause, 2 patients for no improvement in kidney function and, 1 patient because of severe neutropenia.

eGFR: estimate glomerular filtration rate, GC: glucocorticoids, AAV: ANCA associated vasculitis, HD: hemodialysis, IQR: inter-quartile range, DAH: diffuse alveolar hemorrhage, AE: adverse events, PDN: prednisone.

## References

[1] Andrews M., Edmunds M., Campbell A., Walls J., Feehally J. (1990). Systemic vasculitis in the 1980s—Is there an increasing incidence of Wegener’s granulomatosis and microscopic polyarteritis?. J. R. Coll. Physicians Lond..

[2] Watts R.A., Hatemi G., Burns J.C., Mohammad A.J. (2022). Global epidemiology of vasculitis. Nat. Rev. Rheumatol..

[3] Nakazawa D., Masuda S., Tomaru U., Ishizu A. (2019). Pathogenesis and therapeutic interventions for ANCA-associated vasculitis. Nat. Rev. Rheumatol..

[4] McGregor J.G., Hogan S.L., Hu Y., Jennette C.E., Falk R.J., Nachman P.H. (2012). Glucocorticoids and relapse and infection rates in anti-neutrophil cytoplasmic antibody disease. Clin. J. Am. Soc. Nephrol..

[5] Hellmich B., Sanchez-Alamo B., Schirmer J.H., Berti A., Blockmans D., Cid M.C., Holle J.U., Hollinger N., Karadag O., Kronbichler A. (2024). EULAR recommendations for the management of ANCA-associated vasculitis: 2022 update. Ann. Rheum. Dis..

[6] Jayne D.R., Bruchfeld A.N., Harper L., Schaier M., Venning M.C., Hamilton P., Burst V., Grundmann F., Jadoul M., Szombati I. (2017). Randomized Trial of C5a Receptor Inhibitor Avacopan in ANCA-Associated Vasculitis. J. Am. Soc. Nephrol..

[7] Merkel P.A., Niles J., Jimenez R., Spiera R.F., Rovin B.H., Bomback A., Pagnoux C., Potarca A., Schall T.J., Bekker P. (2020). Adjunctive Treatment With Avacopan, an Oral C5a Receptor Inhibitor, in Patients With Antineutrophil Cytoplasmic Antibody–Associated Vasculitis. ACR Open Rheumatol..

[8] Xiao H., Schreiber A., Heeringa P., Falk R.J., Jennette J.C. (2007). Alternative Complement Pathway in the Pathogenesis of Disease Mediated by Anti-Neutrophil Cytoplasmic Autoantibodies. Am. J. Pathol..

[9] Mathern D.R., Heeger P.S. (2015). Molecules Great and Small. Clin. J. Am. Soc. Nephrol..

[10] Renner B., Tong H.H., Laskowski J., Jonscher K., Goetz L., Woolaver R., Hannan J., Li Y.X., Hourcade D., Pickering M.C. (2016). Annexin A2 Enhances Complement Activation by Inhibiting Factor H. J. Immunol..

[11] Noris M., Caprioli J., Bresin E., Mossali C., Pianetti G., Gamba S., Daina E., Fenili C., Castelletti F., Sorosina A. (2010). Relative Role of Genetic Complement Abnormalities in Sporadic and Familial aHUS and Their Impact on Clinical Phenotype. Clin. J. Am. Soc. Nephrol..

[12] Teixeira J.E., Costa R.S., Lachmann P.J., Würzner R., Barbosa J.E. (2003). CR1 stump peptide and terminal complement complexes are found in the glomeruli of lupus nephritis patients. Clin. Exp. Immunol..

[13] Ichida S., Yuzawa Y., Okada H., Yoshioka K., Matsuo S. (1994). Localization of the complement regulatory proteins in the normal human kidney. Kidney Int..

[14] Hsu S.I.H., Couser W.G. (2003). Chronic Progression of Tubulointerstitial Damage in Proteinuric Renal Disease Is Mediated by Complement Activation. J. Am. Soc. Nephrol..

[15] Sheerm N.S., Risley P., Abe K., Tang Z., Wong W., Lin T., Sacks S.H. (2008). Synthesis of complement protein C3 in the kidney is an important mediator of local tissue injury. FASEB J..

[16] Farrar C.A., Zhou W., Lin T., Sacks S.H. (2006). Local extravascular pool of C3 is a determinant of postischemic acute renal failure. FASEB J..

[17] Villacorta J., Diaz-Crespo F., Acevedo M., Cavero T., Guerrero C., Praga M., Fernandez-Juarez G. (2016). Circulating C3 levels predict renal and global outcome in patients with renal vasculitis. Clin. Rheumatol..

[18] Gou S.J., Yuan J., Chen M., Yu F., Zhao M.H. (2013). Circulating complement activation in patients with anti-neutrophil cytoplasmic antibody-associated vasculitis. Kidney Int..

[19] Haas M., Eustace J.A. (2004). Immune complex deposits in ANCA-associated crescentic glomerulonephritis: A study of 126 cases. Kidney Int..

[20] Xing G.Q., Chen M., Liu G., Heeringa P., Zhang J.J., Zheng X., E J., Kallenberg C.G., Zhao M.H. (2009). Complement Activation Is Involved in Renal Damage in Human Antineutrophil Cytoplasmic Autoantibody Associated Pauci-Immune Vasculitis. J. Clin. Immunol..

[21] Mazzariol M., Manenti L., Vaglio A. (2023). The complement system in antineutrophil cytoplasmic antibody-associated vasculitis: Pathogenic player and therapeutic target. Curr. Opin. Rheumatol..

[22] Chen S.F., Wang H., Huang Y.M., Li Z.Y., Wang S.X., Yu F., Zhao M.H., Chen M. (2015). Clinicopathologic Characteristics and Outcomes of Renal Thrombotic Microangiopathy in Anti-Neutrophil Cytoplasmic Autoantibody-Associated Glomerulonephritis. Clin. J. Am. Soc. Nephrol..

[23] Lucientes-Continente L., Fernández-Juárez G., Márquez-Tirado B., Jiménez-Villegas L., Acevedo M., Cavero T., Cámara L.S., Draibe J., Anton-Pampols P., Caravaca-Fontán F. (2024). Complement alternative pathway determines disease susceptibility and severity in antineutrophil cytoplasmic antibody (ANCA)-associated vasculitis. Kidney Int..

[24] Xiao H., Dairaghi D.J., Powers J.P., Ertl L.S., Baumgart T., Wang Y., Seitz L.C., Penfold M.E., Gan L., Hu P. (2014). C5a receptor (CD88) blockade protects against MPO-ANCA GN. J. Am. Soc. Nephrol..

[25] Jayne D.R.W., Merkel P.A., Schall T.J., Bekker P., ADVOCATE Study Group (2021). Avacopan for the Treatment of ANCA-Associated Vasculitis. N. Engl. J. Med..

[26] Chalkia A., Jayne D. (2024). ANCA-associated vasculitis-treatment standard. Nephrol. Dial. Transplant..

[27] Zonozi R., Aqeel F., Le D., Cortazar F.B., Thaker J., Ramirez M.J.Z., Cortes S.E.S., Attieh R.M., Chung M., Bulbin D.H. (2024). Real-World Experience With Avacopan in Antineutrophil Cytoplasmic Autoantibody-Associated Vasculitis. Kidney Int. Rep..

[28] Kataoka H., Tomita T., Nakanowatari M., Kondo M., Mukai M. (2023). Gradual increase of avacopan dose with concomitant ursodeoxycholic acid use may help avoid the risk of C5a receptor inhibitor-induced liver injury in antineutrophil cytoplasmic antibody-associated vasculitis. Mod. Rheumatol. Case Rep..

[29] Morimoto N., Mori T., Shioji S., Watanabe H., Sakai K., Mori K., Yamamura A., Hanioka A., Akagi Y., Fujiki T. (2023). Thrombocytopenia during avacopan administration: A case report. Int. J. Rheum. Dis..

[30] Barr B., Cheema K., Fifi-Mah A., Garner S., Girard L.P. (2024). Use of Avacopan in Patients With Antineutrophil Cytoplasmic Antibody-Associated Vasculitis and Estimated Glomerular Filtration Rate <15 mL/min per 1.73 m^2^. Kidney Int. Rep..

[31] Sachez-Alamo B., Moi L., Bajema I., Berden A., Flossmann O., Hruskova Z., Jayne D., Wester-Trejo M., Wallquist C., Westman K. (2024). Long-term outcome of kidney function in patients with ANCA-associated vasculitis. Nephrol. Dial. Transplant..

[32] Cortazar F.B., Niles J.L., Jayne D.R., Merkel P.A., Bruchfeld A., Yue H., Schall T.J., Bekker P., Peh C.A., Chakera A. (2023). Renal Recovery for Patients with ANCA-Associated Vasculitis and Low eGFR in the ADVOCATE Trial of Avacopan. Kidney Int. Rep..

[33] Zimmermann J., Sonnemann J., Jabs W.J., Schönermarck U., Vielhauer V., Bieringer M., Schneider U., Kettritz R., Schreiber A. (2024). Avacopan in Anti-Neutrophil Cytoplasmic Autoantibodies–Associated Vasculitis in a Real-World Setting. Kidney Int. Rep..

[34] Draibe J., Espigol-Frigolé G., Cid M.C., Prados M.C., Guillén E., Villacorta J., Vega C., Martins J., daSilva I., Martin-Gomez M.A. (2024). The real-world use and effectiveness of avacopan in routine practice for the treatment of ANCA vasculitis. First experiences in Spain. Rheumatology.

[35] Strand V., Jayne D.R., Horomanski A., Yue H., Bekker P., Merkel P.A., Peh C.A., Chakera A., Cooper B., Kurtkoti J. (2023). The impact of treatment with avacopan on health-related quality of life in antineutrophil cytoplasmic antibody-associated vasculitis: A post-hoc analysis of data from the ADVOCATE trial. Lancet Rheumatol..

[36] Floege J., Jayne D.R., Sanders J.S.F., Tesar V., Balk E.M., Gordon C.E., Adam G., Tonelli M.A., Cheung M., Earley A. (2024). Executive summary of the KDIGO 2024 Clinical Practice Guideline for the Management of ANCA–Associated Vasculitis. Kidney Int..

[37] Geetha D., Dua A., Yue H., Springer J., Salvarani C., Jayne D., Merkel P. (2024). Efficacy and safety of avacopan in patients with ANCA-associated vasculitis receiving rituximab in a randomised trial. Ann. Rheum. Dis..

[38] Falde S.D., Lal A., Cartin-Ceba R., Mertz L.E., Fervenza F.C., Zand L., Koster M.J., Warrington K.J., Lee A.S., Aslam N. (2024). Treatment of Antineutrophil Cytoplasmic Antibody–Associated Vasculitis With Diffuse Alveolar Hemorrhage With Avacopan. ACR Open Rheumatol..

[39] Chalkia A., Flossmann O., Jones R., Nair J.R., Simpson T., Smith R., Willcocks L., Jayne D. (2024). Avacopan for ANCA-associated vasculitis with hypoxic pulmonary haemorrhage. Nephrol. Dial. Transplant..

[40] Hakroush S., Tampe B. (2023). Tailored Use of Avacopan in a Case With Refractory Antineutrophil Cytoplasmic Antibody-Associated Renal Vasculitis and Concominant Complement System Activation. Kidney Int. Rep..

[41] Patel N.J., Jayne D.R., Merkel P.A., Bekker P., Zhang Y., McDowell P.J., Johal J., Heaney L.G., Murrell D., Stone M.N. (2023). The Glucocorticoid Toxicity Index-Metabolic Domains, an abridged version of the Glucocorticoid Toxicity Index: Post-hoc analysis of data from the ADVOCATE trial. Lancet Rheumatol..

[42] Duru N., Van Der Goes M.C., Jacobs J.W.G., Andrews T., Boers M., Buttgereit F., Caeyers N., Cutolo M., Halliday S., Da Silva J.A.P. (2013). EULAR evidence-based and consensus-based recommendations on the management of medium to high-dose glucocorticoid therapy in rheumatic diseases. Ann. Rheum. Dis..

[43] van Leeuwen J.R., Bredewold O.W., van Dam L.S., Werkman S.L., Jonker J.T., Geelhoed M., Langeveld A.P., Remmelts H.H., van den Broecke M.M., Ray A. (2022). Compassionate Use of Avacopan in Difficult-to-Treat Antineutrophil Cytoplasmic Antibody–Associated Vasculitis. Kidney Int. Rep..

[44] Osman M., Cohen Tervaert J.W., Pagnoux C. (2023). Avacopan for the treatment of ANCA-associated vasculitis: An update. Expert. Rev. Clin. Immunol..

[45] Gabilan C., Belliere J., Moranne O., Pfirmann P., Samson M., Delattre V., Thoreau B., Gueutin V., Boyer A., Leurs A. (2024). Avacopan for anti-neutrophil cytoplasm antibodies-associated vasculitis: A multicentre real-world study. Rheumatology.

[46] Walsh M., Merkel P.A., Peh C.A., Szpirt W.M., Puéchal X., Fujimoto S., Hawley C.M., Khalidi N., Floßmann O., Wald R. (2020). Plasma Exchange and Glucocorticoids in Severe ANCA-Associated Vasculitis. N. Engl. J. Med..

[47] Okubo A., Fukui S., Tanigawa M., Kojima K., Sumiyoshi R., Koga T., Shojinaga S., Sakamoto R., Nakashima M., Kawakami A. (2024). Improved Hearing Impairment of Granulomatosis with Polyangiitis Treated with Rituximab and Avacopan without Glucocorticoids. Intern. Med..

[48] Abrantes A.M., Montaño-Tapia L., Isenberg D. (2023). ANCA-MPO: Is this a useful test?. Rheumatology.

